# Lithium chloride at environmental concentrations impairs microtubule function and promotes genotoxicity in *Allium cepa*

**DOI:** 10.1038/s41598-025-21567-5

**Published:** 2025-10-28

**Authors:** Gabriela Gomes Lima, Carlos Filipe Camilo-Cotrim, Junilson Augusto Paula Silva, Myllena Tolentino Firmino, Natanael Alves Silva, Antônio Sérgio Nakao Aguiar, Marcelino Benvindo-Souza, Leonardo Luiz Borges, Samantha Salomão Caramori, Elisa Flávia Luiz Cardoso Bailão, Luciane Madureira Almeida

**Affiliations:** 1 Laboratório de Biotecnologia, Universidade Estadual de Goiás, BR 153, No. 3105, Fazenda Barreiro do Meio – Henrique Santillo Campus, P.O. Box 459, Anápolis, GO 75132-400 Brazil; 2 Laboratório de Novos Materiais, Universidade Evangélica de Goiás, Anápolis, GO 75083-515 Brazil; 3 Grupo de Química Teórica e Estrutural de Anápolis, Universidade Estadual de Goiás, Anápolis, GO 75132-903 Brazil; 4 Escola de Ciências Médicas e da Vida, Pontifícia Universidade Católica de Goiás, Goiânia, GO Brazil

**Keywords:** Aneugenicity, Cytotoxicity, Ecotoxicology, Emerging contaminant, Microtubule disruption, Oxidative stress, Environmental sciences, Environmental impact

## Abstract

The growing demand for lithium, driven by the energy transition and widespread use of rechargeable batteries, has raised concerns about its environmental release. This study assessed the toxicological effects of lithium chloride (LiCl) at environmentally relevant concentrations using the *Allium cepa* bioassay. While lithium’s genotoxicity at high concentrations is known, its effects at levels typical of aquatic systems (up to 4 mg/L) remain poorly understood. A set of biomarkers was applied to evaluate cytotoxicity, genotoxicity, oxidative stress, and in silico molecular interactions. LiCl exposure significantly reduced the mitotic index, indicating cytotoxic effects via impaired cell division. Increased chromosomal aberrations and nuclear abnormalities were observed, particularly at 4 mg/L, suggesting genotoxicity. However, the Comet assay revealed minimal DNA strand breaks, pointing to an aneugenic mechanism likely caused by mitotic spindle disruption rather than clastogenic effects. Cell cycle analysis showed reduced metaphase and increased anaphase frequencies, reinforcing the hypothesis of chromosomal missegregation. In silico modeling demonstrated strong interactions between Li^+^ ions and tubulin, potentially affecting spindle stability. Additionally, altered superoxide dismutase (SOD) activity indicated oxidative stress involvement. Overall, lithium at environmentally realistic concentrations induces cytotoxic and genotoxic effects in *A. cepa*, primarily through aneugenic mechanisms linked to oxidative stress and microtubule disruption.

## Introduction

Various human activities have introduced trace amounts of metals into water and soil, altering ecosystems and impacting the health and behavior of numerous taxa^1–4^. Among these metals, lithium is of particular concern due to its low mass and high water solubility, facilitating its dispersion and contamination of aquatic environments. Precipitation and surface runoff can carry lithium into rivers, streams, lakes, and creeks, enabling its transport over long distances from its original source and increasing its environmental presence. Moreover, lithium’s high reactivity prevents it from existing in its elemental form in nature; instead, it is found in compounds such as lithium carbonate, lithium chloride, spodumene, and lepidolite^5–7^.

The rising technological demand for lithium has made it an emerging environmental contaminant of concern, particularly due to its crucial role in the energy transition and the advancement of green technologies. Lithium-ion batteries (LIBs), which are widely used in cell phones, laptops, tablets, cameras, and other portable electronic devices, as well as in electric and hybrid vehicles and renewable energy storage systems, represent the primary application of lithium^8–10^. Global lithium production is estimated at approximately 82,500 metric tons per year, with the LIB industry accounting for 74% of total lithium consumption^11^.

LIBs are widely recognized for their efficiency in converting chemical energy into electrical energy, offering significant advantages such as cost reduction and increased production capacity^12^. Their key attributes—including low weight (as lithium is the lightest metal in the periodic table), high energy density, long service life, low self-discharge rate, and lower toxicity compared to other battery types, such as nickel–cadmium and nickel-metal hydride have established LIBs as a preferred solution for energy storage^13^. Due to these benefits and the increasing global demand for electronic devices, LIB production has expanded significantly, accounting for approximately 60% of the portable energy market^14^.

However, the increasing production of LIBs is accompanied by a significant rise in lithium-containing waste, contributing to environmental contamination. Despite the widespread use of lithium, its ecological effects remain poorly understood. Studies have reported various adverse impacts of lithium on aquatic organisms, including developmental inhibition^15^; cellular and metabolic alterations^16–18^; growth and reproductive inhibition^19^; increased oxidative stress^20^; and metabolic, immunological, and histopathological changes^21^. Furthermore, lithium has been associated with behavioral and metamorphic changes^22^.

Lithium’s genotoxic effects have been recently demonstrated in *Allium cepa* assays^23^. In onions, lithium toxicity was directly linked to oxidative stress, leading to interactions with DNA and resulting in a high rate of genotoxic damage^23^. Anatomical alterations were also observed, including epidermal and cortical damage, nuclear flattening, and other structural abnormalities^23^. High concentrations of lithium carbonate (25 mg/L, 50 mg/L, and 100 mg/L) were tested due to the plant’s tolerance to lithium. However, lithium concentrations in water resources are generally lower, ranging from 0.18 mg/L to 14 mg/L^5,24,25^.

Thus, while high lithium concentrations have shown genotoxic potential in plants, few studies have examined its toxicity at environmentally relevant concentrations. Therefore, this study aims to investigate whether lithium at environmentally relevant levels (up to 4 mg/L) exhibits toxic effects on *Allium cepa*. This model, widely recognized for its enzymatic system’s similarity to mammalian detoxification mechanisms, enables the identification of cytogenetic and physiological effects in eukaryotic cells^23^.

## Material and methods

### Material acquisition

Onion bulbs were obtained in the Centro Estadual de Abastecimento (CEASA) of Anápolis, Goiás, Brazil. The lithium chloride (CAS No. 7447-41-8) was obtained from Sigma-Aldrich. Lithium concentrations were selected based on environmental occurrence and regulatory standards. Natural freshwater bodies have been reported to contain lithium levels of up to 14 mg/L, while Brazilian legislation^26^ allows a maximum of 2.5 mg/L. Accordingly, the study tested lithium-ion concentrations of 0.16, 0.41, 1.6, and 4 mg/L, corresponding to lithium chloride concentrations of 1, 2.5, 10, and 25 mg/L, respectively (Table 1).

**Table 1:** Tested concentrations of lithium chloride (LiCl) and the corresponding lithium-ion (Li^+^) concentrations (mg/L) used in the *Allium cepa* assays. Negative control (NC) consisted of purified water only. Treatments I–IV represent the experimental groups exposed to increasing concentrations of LiCl.

Treatments	Concentration (mg/L)	
	Li^+^	LiCl
Negative control: NC	0	0
I	0.16	1.00
II	0.41	2.50
III	1.60	10.0
IV	4.00	25.00

### Assessment of cytotoxic and genotoxic effects

Cytotoxic and genotoxic effects were assessed using a protocol adapted from Alam^27^. *Allium cepa* bulbs (type 2, 35–50 mm diameter) were commercially sourced based on size uniformity and structural integrity. Bulbs were germinated in purified water for seven days in a B.O.D. incubator at 23 °C, with daily water renewal to maintain optimal conditions. Following germination, bulbs were exposed to lithium chloride solutions at 1, 2.5, 10, and 25 mg/L. Purified water was used as negative control. Each treatment group consisted of 15 bulbs (n = 15), with three independent replicates of five bulbs each. Bulbs were incubated in treatment solutions for 48 h, with daily renewal. For cytogenetic analyses, 1,000 cells were scored per bulb, totaling 15,000 cells per treatment group. Post-exposure, root tips were excised and fixed in Carnoy’s solution (ethanol: acetic acid, 3:1) for 24 h, then stored in 70% ethanol at 4 °C until analysis. Roots were rinsed twice with purified water and treated with acetic acid (~ 2 min per wash).

Hydrolysis was performed in 1 N HCl at 60 °C for 8–10 min, then rinsed with distilled water. Samples were stained in Schiff’s reagent at 23 °C for 45 min, followed by two additional water washes. Roots were treated with 45% acetic acid for 1 min for slide preparation. Three roots per bulb were selected, and the most intensely stained meristematic regions were minced. A 2% acetic carmine drop was added, and the slides were gently heated to dissociate the tissue. Two slides were prepared per bulb. Coverslips were removed using liquid nitrogen, and slides were dried overnight at 23 °C. The following day, 50 μL of Entellan was applied, and the slides were sealed with a new coverslip and incubated at 25 °C overnight. Finally, the slides were cleaned with 70% ethanol. Microscopic evaluation was performed under light microscopy at 40 × and 100 × magnifications. For each bulb, approximately 1000 cells were analyzed, totaling 15 bulbs per experimental group. In this count, the frequency of dividing cells was recorded for mitotic index calculation, while chromosomal aberrations and nuclear abnormalities were scored to assess genotoxicity and mutagenicity. The following formulas were used for data analysis:

(i) The Mitotic index (MI) was calculated with the following formula:

$$\text{MI per replica }(\text{\%})=\left(\frac{\#\text{ of cells in division per treatment}}{\#\text{ of cells counted per treatment}}\right)*100$$

(ii) MI inhibition/stimulation is calculated by the relative difference between the control and the treatment, normalized by the control’s MI, multiplied by -100, where positive values indicate the stimulation percentage and negative ones the inhibition percentage. The applied formula was:

$$\text{MI inhibition/stimulation per replica }\left(\text{\%}\right)\text{ = }\frac{\text{control MI - treatment MI}}{\text{control MI}}\text{*100}$$

(iii) % of mitotic phases per replica, calculated by:

$$\text{Phases per replica }(\text{\%})=\left(\frac{\text{particular phase per treatment}}{\#\text{ of cells in division per treatment}}\right)*100$$

(iv) Chromosomal aberration (CA) index, calculated by:

$$\text{CA per replica }(\text{\%})=\left(\frac{\text{total CA}}{\#\text{ of cells counted per treatment}}\right)*100$$

(v) Nuclear abnormality (NA) index, calculated by:

$$\text{NA per replica }(\text{\%})=\left(\frac{\text{total NA}}{\#\text{ of cells counted per treatment}}\right)*100$$

(vi) Micronucleus (Mn) index, calculated by:

$$\text{Mn per replica }(\text{\%})=\left(\frac{\text{total Mn}}{\#\text{ of cells counted per treatment}}\right)*100$$

### Comet assay

The comet assay assessed the genotoxicity of lithium chloride in *Allium cepa*, following a modified protocol based on Gichner et al.^28^ and Türkoğlu^29^. Post-treatment, root tips were transferred to Petri dishes containing 60 μL of cold 400 mM Tris buffer (pH 7.5) per set of three tips and kept on ice. Roots were gently minced with a scalpel, and the dishes were inclined over ice to facilitate nuclear release and accumulation. Sixty microliters of the suspension were mixed with 240 μL of 0.1% low-melting-point agarose (40 °C) and layered onto slides pre-coated with 1.5% standard agarose. Preparations were covered with coverslips and kept at 4 °C for 3 min to solidify. Coverslips were then removed, and slides were incubated on ice for 1 h. After washing to remove salts, slides were immersed in alkaline electrophoresis buffer (EDTA and NaOH) for 20 min. Electrophoresis was performed in the dark for 30 min at 25 V and 300 mA. Slides were then neutralized three times in 0.4 M Tris buffer (pH 7.5) for 5 min each, rinsed with distilled water, and fixed with absolute ethanol. After drying, DNA was stained with 100 μL of Diamond™ fluorescent dye. Comets were visualized using a fluorescence microscope (Axio Imager® A2, Carl Zeiss AG, Germany) with a 510–560 nm excitation filter, a 590 nm emission filter, and a 20 × objective. A single examiner performed all analyses. DNA damage was quantified with TriTek CometScore 2.0 software (http://rexhoover.com/index.php?id=cometscore), based on 100 nucleoids per sample. The following parameters were evaluated: tail DNA percentage (%DNA), tail length, and Olive tail moment.

### Antioxidant enzyme activity

The effects of LiCl on antioxidant defense were assessed by measuring the inhibition of superoxide dismutase (SOD) activity in root meristematic cells, following the method of Srivastava and Singh^30^ with modifications. Sets of three root tips were transferred after treatment to Petri dishes containing 60 µL of phosphate buffer (0.05 M, pH 7.0) and excised on ice to facilitate enzyme extraction. The resulting supernatant was collected for enzymatic analysis. SOD activity was quantified using the Superoxide Dismutase Activity Assay Kit (Sigma-Aldrich, No. 1216-1503), according to the manufacturer’s instructions, and absorbance was measured at 450 nm with a microplate reader.

### Molecular modeling

To investigate how the Li^+^ cation interacts with tubulin, a complexed structure of this protein with the co-crystallized ligand nocodazole (PDB ID: 7Z2P) was used^31^. This antineoplastic agent interacts directly with the side chains of Ala316B, Leu255B, Ile378B, Asn167B, and Glu200B. These residues were then subjected to modeling analyses with the Li^+^ cation to verify possible interactions with these residues.

Based on this pattern of intermolecular interactions, the molecular structures of these amino acid residues were optimized using density functional theory (DFT)^32,33^, as implemented in the Gaussian 16 software package. The theoretical calculations employed the highly parameterized empirical exchange–correlation functional M06-2X combined with the diffuse and polarized 6–311++ G(d,p) basis set^34^. Molecular electrostatic potential maps were generated for each residue, and the Li^+^ ion was positioned in the region of the highest electron density within the side chain. The resulting Li^+^–amino acid complexes were then subjected to calculations at the same level of theory, and their molecular topologies were analyzed using Bader’s quantum theory of atoms in molecules (QTAIM)^35,36^. Additionally, binding energy corrections were computed using the counterpoise method to determine which residue(s) interacted most strongly with the Li^+^ cation^37^.

### Statistical analysis

The assumptions of normality and homogeneity of variances were evaluated prior to statistical analysis. When these assumptions were met, a one-way ANOVA was conducted, followed by Tukey’s post-hoc test. In cases where assumptions were violated, the data were transformed using log(x + 0.5) and reassessed. If the transformed data did not meet the assumptions, the nonparametric Kruskal–Wallis test was applied, followed by the Dwass-Steel-Critchlow-Fligner post-hoc test. For the comet assay, the Kruskal–Wallis test was followed by Dunn’s test. Statistical significance was set at *p* < 0.05, and results were expressed as mean ± standard deviation (SD). All statistical analyses were performed using the software Statistica 7^38^.

## Results and discussion

This study evaluated the toxicological effects of lithium chloride at environmentally relevant concentrations using the *Allium cepa* bioassay, a widely accepted model in environmental toxicology. A comprehensive set of biomarkers was employed, including assessments of cytotoxicity, genotoxicity, oxidative stress, and in silico molecular modeling. The integration of these endpoints provided a multi-level perspective on lithium chloride induced cellular and molecular alterations. Given the increasing environmental presence of lithium due to its extensive industrial use, this research highlights the importance of investigating whether environmentally relevant concentrations can harm living organisms and ecosystems.

### Cytotoxicity effects

**Fig. 1:**
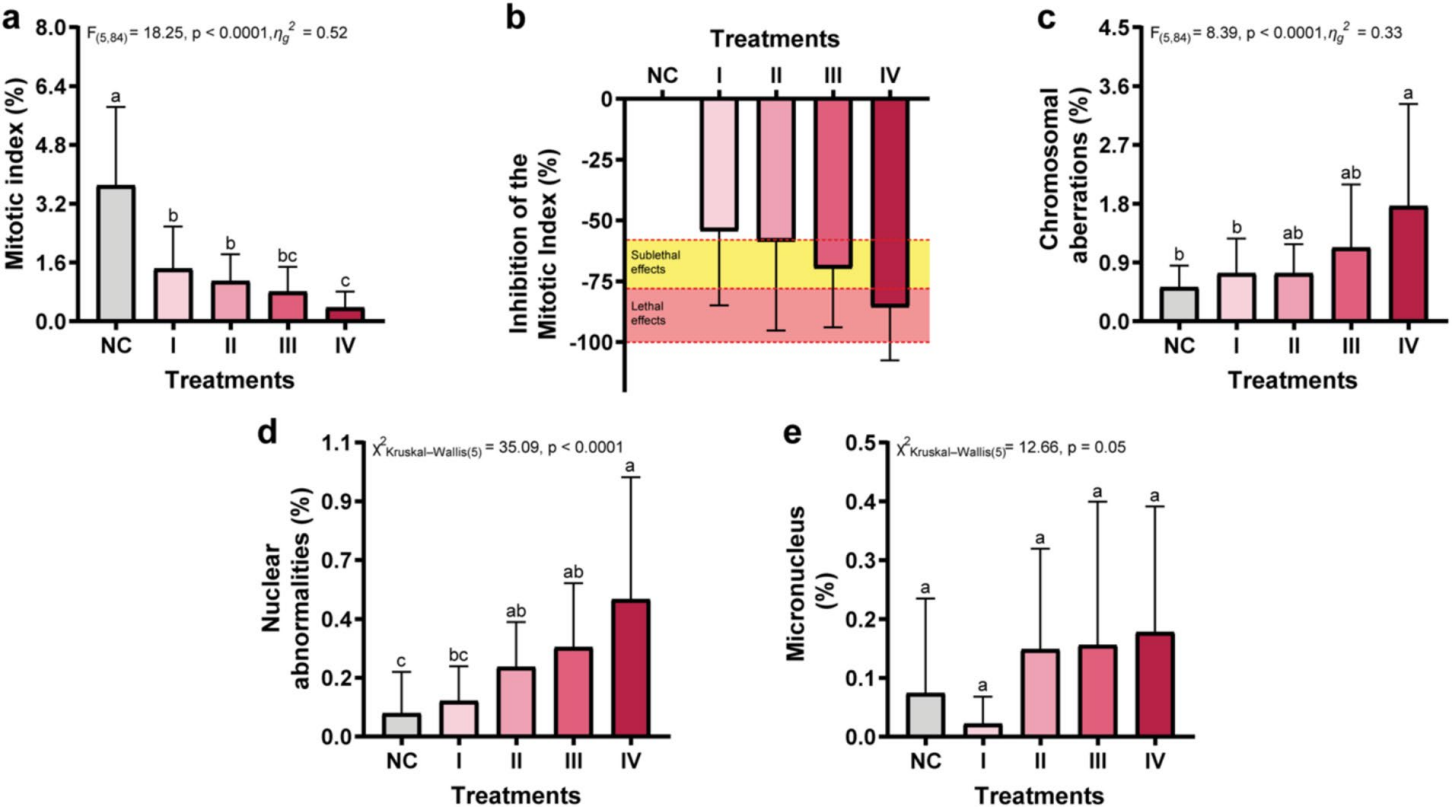
Cytogenetic parameters in *Allium cepa* root meristems exposed to increasing concentrations of lithium chloride (LiCl). (**a**) Mitotic index, indicating the frequency of cell division; (**b**) percentage of mitotic index inhibition compared to the negative control (NC, distilled water); (**c**) chromosomal aberrations (e.g., c-mitosis, anaphase bridges, laggards), reflecting spindle and chromosomal instability; (**d**) nuclear abnormalities, such as lobulated or binucleated nuclei; and (**e**) frequency of micronuclei. LiCl concentrations tested were I. 1 mg/L; II. 2.5 mg/L; III. 10 mg/L; and IV. 25 mg/L, corresponding to I. 0.16 mg/L, II. 0.41 mg/L, III. 1.6 mg/L, and IV. 4 mg/L of lithium-ion. Data are presented as mean ± standard error (SE). Statistical analyses were performed after verification of normality (Shapiro–Wilk test) and homogeneity of variance (Levene’s test). Group comparisons were made by one-way ANOVA followed by Tukey’s post hoc test. In the graphs, means followed by the same letter do not differ significantly at p < 0.05.

The initial assessment of lithium chloride cytotoxicity in *A. cepa* root cells was conducted using the mitotic index (MI) as a biomarker of cell proliferation. Our results revealed a statistically significant variation in MI across treatment groups (F_(5,84)_ = 18.25; p < 0.0001; η^2^g = 0.52), with an evident concentration-dependent decline following LiCl exposure (Fig. 1a). Mitotic activity inhibition remained below 20% in all treated groups compared to the negative control, suggesting a pronounced anti-mitotic effect (Fig. 1b). Treatment III (1.6 mg/L) was classified as sublethal, while Treatment IV (4 mg/L) exhibited lethal effects. The concentration-dependent reduction in MI reflects a marked suppression of cell division in *A. cepa* root meristem cells. This mitodepressive response, which intensified at higher concentrations, highlights the cytotoxic potential of lithium chloride at the cellular level.

These findings are consistent with previous studies that have reported lithium-induced cytotoxicity in plant systems. For instance, Kuloğlu et al.^23^ demonstrated that exposure to lithium carbonate led to a dose-dependent reduction in the mitotic index and increased oxidative stress in *A. cepa* meristematic cells, reinforcing lithium’s capacity to impair essential cellular functions in plants. Similarly, lithium’s cytotoxic effects have been documented in mammalian systems. Pastor et al.^39^ reported that lithium chloride induced dose-dependent cytotoxicity in Chinese hamster ovary cells, manifesting as mitotic abnormalities, including multipolar divisions and chromosomal lagging. Given these cytotoxic effects, we further investigated whether lithium chloride also exerts genotoxic activity by evaluating chromosomal and nuclear abnormalities in *A. cepa* root meristem cells.

### Genotoxic effects

**Fig. 2:**
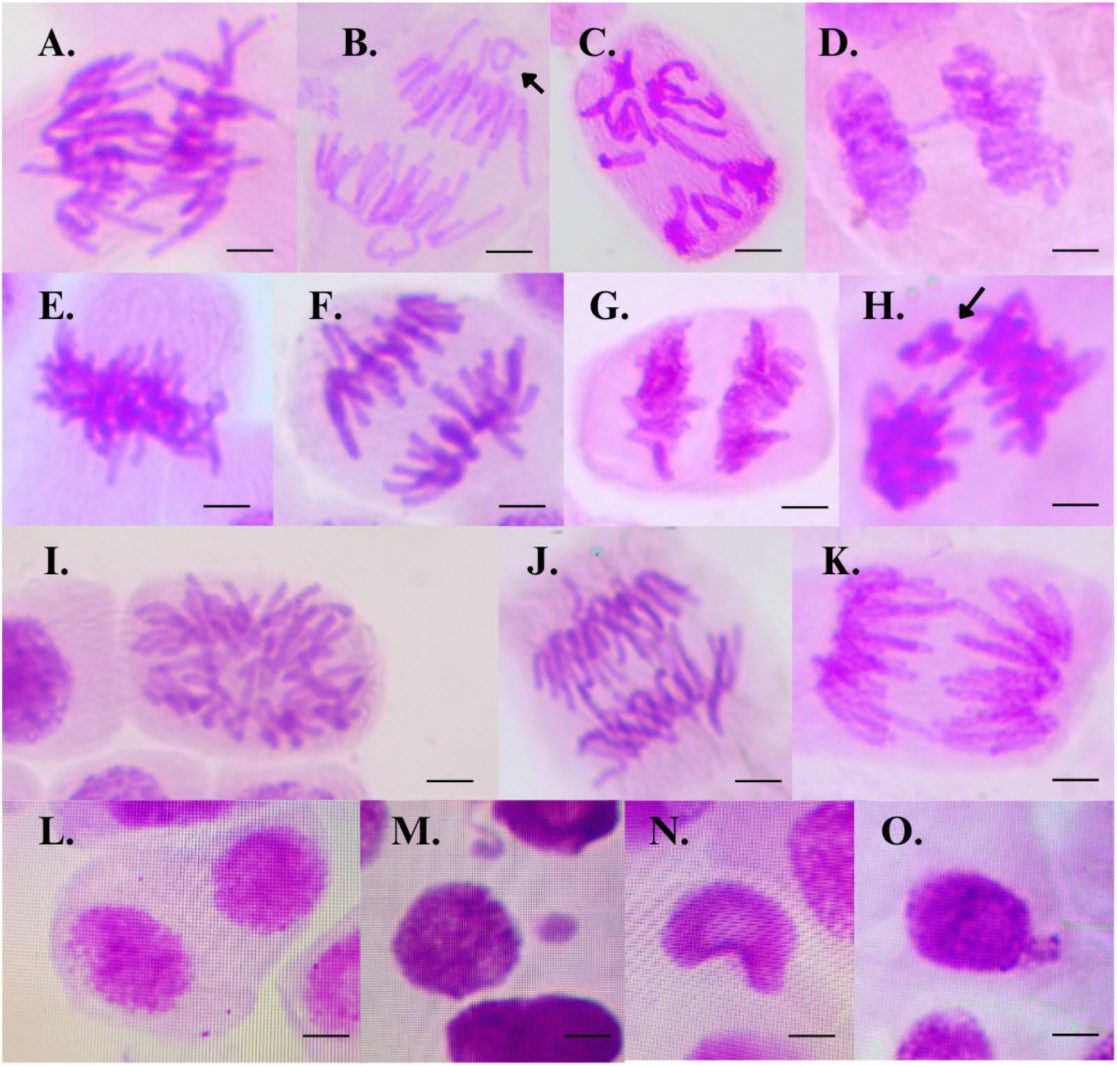
Representative chromosomal and nuclear aberrations in *Allium cepa* root meristem cells exposed to environmentally relevant concentrations of lithium chloride (LiCl). Observed abnormalities include: (**A**) chromosome lagging, (**B**) chromosome loss, (**C**) c-mitosis without polyploidy, (**D**) chromosome bridge, (**E**) polyploid metaphase, (**F**) vagrant chromosome, (**G**) chromosome stickiness, (**H**) chromosome loss, (**I**) c-mitosis with polyploidy, (**J**) chromosome bridge, (**K**) multiple chromosome bridges, (**L**) binucleated cell, (**M**) micronucleus, (**N**) lobed nucleus, and (**O**) nuclear bud. Images were obtained by light microscopy at 40 × and 100 × magnifications. Approximately 1,000 cells per bulb were analyzed, with 15 bulbs per treatment group. Scale bar: 10 μm.

Exposure to lithium chloride resulted in a significant increase in chromosomal aberrations, with the most pronounced effects observed in Treatment IV (F_(5,84)_ = 8.39; p < 0.0001; η^2^g = 0.33) (Fig. 1c). Moreover, the frequency of nuclear abnormalities exhibited a clear concentration-dependent pattern (χ^2^ Kruskal–Wallis_(5)_ = 35.09; p < 0.0001), reaching its peak in the highest exposure group (Fig. 1d). These findings indicate that lithium chloride exerts genotoxic and mutagenic effects at the chromosomal level, particularly at the concentration of 4.00 mg/L, as evidenced by the increased prevalence of mitotic and nuclear alterations (Figs. 1c and 2). In contrast, the incidence of micronuclei did not significantly differ from the negative control across all treatment groups (Fig. 1e), suggesting that lithium chloride does not induce clastogenic damage to DNA at the concentrations tested. To further investigate the nature and extent of DNA damage, particularly at the molecular level, we next employed the Comet assay to assess primary DNA strand breaks in individual cells.

### Comet assay

**Fig. 3:**
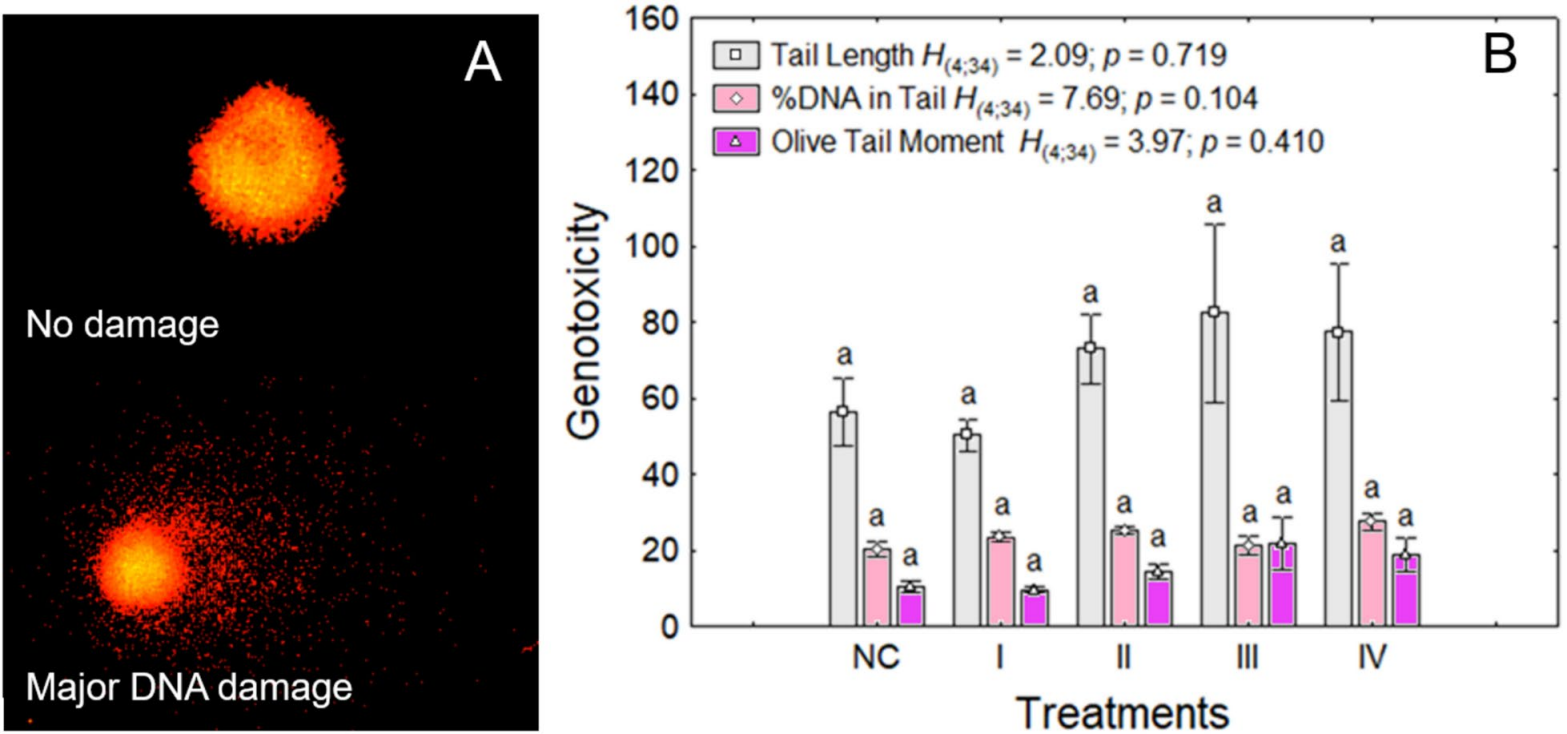
Representative micrographs and quantification of DNA damage in *Allium cepa* root meristem cells subjected to the comet assay after exposure to environmentally relevant concentrations of lithium chloride (LiCl). (**A**) Representative nuclei stained with Diamond™ dye showing different classes of DNA migration: intact nucleus (no tail) and nuclei with increasing comet tail formation indicative of DNA strand breaks. (**B**) Quantitative analysis of comet assay parameters: %DNA in the tail, tail length (µm), and Olive tail moment, each reflecting distinct aspects of DNA damage. For each treatment group, 100 nuclei per bulb were analyzed, with 5 bulbs per group (n = 500 nuclei). LiCl concentrations: I = 1 mg/L (Li^+^ 0.16 mg/L); II = 2.5 mg/L (Li^+^ 0.41 mg/L); III = 10 mg/L (Li^+^ 1.6 mg/L); IV = 25 mg/L (Li^+^ 4 mg/L). NC = negative control (distilled water). Data are expressed as mean ± standard error (SE). Statistical comparisons were performed using the Kruskal–Wallis test followed by Dunn’s post hoc test; groups sharing the same letter do not differ significantly (p < 0.05).

To determine whether the genotoxic effects of lithium chloride were associated with clastogenic mechanisms, as previously described in the literature for high lithium concentrations via oxidative stress pathways^23^, we subjected *Allium cepa* root cells to the comet assay. This approach detected potential DNA strand breaks and fragmentation, which are hallmark indicators of clastogenic damage. The comet assay was conducted to assess genotoxicity in *Allium cepa* root cells (Fig. 3a, Table 2). No significant differences (p > 0.05) were observed among the lithium chloride treated groups across any of the evaluated parameters (Fig. 3b, Table 2). The low level of DNA fragmentation observed in the comet assay suggests that lithium chloride’s genotoxic effects in *Allium cepa* are predominantly aneugenic rather than clastogenic. Aneugenic agents typically interfere with the spindle apparatus, leading to chromosomal missegregation without directly damaging DNA. If this hypothesis holds, one would expect disruptions in the normal progression of mitosis, particularly in the distribution of cells across the different phases of cell division. Therefore, to further explore the potential aneugenic action of lithium chloride, we next examined the frequency of cells in each mitotic phase.

**Table 2:** Genotoxic damage frequency of lithium chloride (LiCl) in *Allium cepa*. LiCl concentrations: I = 1 mg/L (Li^+^ 0.16 mg/L); II = 2.5 mg/L (Li^+^ 0.41 mg/L); III = 10 mg/L (Li^+^ 1.6 mg/L); IV = 25 mg/L (Li^+^ 4 mg/L). NC = negative control (distilled water). Data are expressed as mean ± standard error (SE). Statistical comparisons were performed using the Kruskal–Wallis test followed by Dunn’s post hoc test; groups sharing the same letter do not differ significantly (p < 0.05).

Treatments	Mean ± standard deviation of comet assay parameters		
	%DNA in tail	Tail length	Olive tail moment
CN	20.20 ± 5.15^a^	56.25 ± 22.89^a^	10.44 ± 4.15^a^
I	23.80 ± 3.47^a^	56.87 ± 21.55^a^	11.09 ± 4.74^a^
II	24.38 ± 3.04^a^	60.20 ± 24.98^a^	12.03 ± 4.81^a^
III	21.06 ± 7.85^a^	90.32 ± 71.64^a^	23.71 ± 21.37^a^
IV	27.58 ± 6.37^a^	77.33 ± 50.42^a^	18.82 ± 12.98^a^
Statistic	*H*_*(5;40)*_ = 6.73; *p* = 0.241	*H*_*(5;40)*_ = 1.85; p = 0.869	*H*_*(5;40)*_ = 3.33; p = 0.648

### Cell cycle phases

**Fig. 4:**
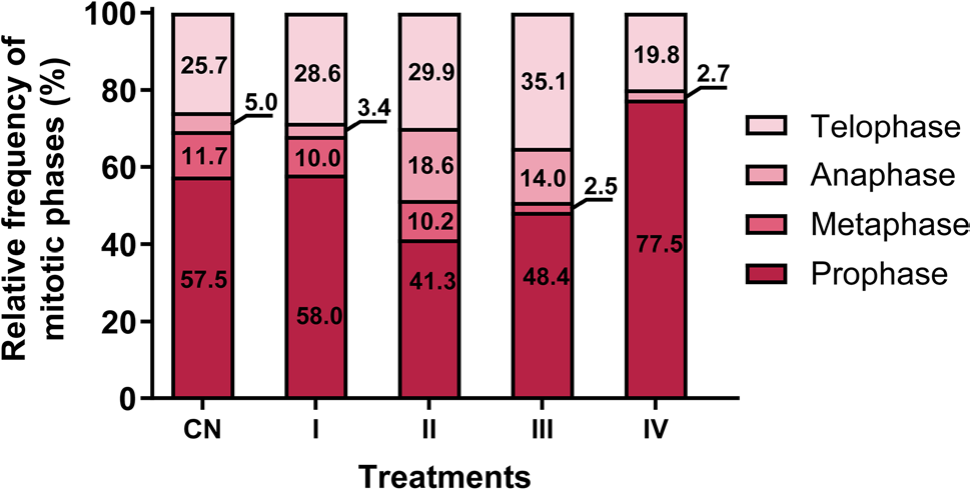
Frequency of cell cycle phases. LiCl concentrations. **I.** 1 mg/L; **II.** 2.5 mg/L; **III.** 10 mg/L and **IV**. 25 mg/L that corresponded to **I.** 0.16 mg/L; **II.** 0.41 mg/L; **III.**1.6 mg/L and **IV.** 4 mg/L of lithium-ion. NC negative control (water). Data are presented as mean ± standard error (SE). Statistical comparisons were performed by one-way ANOVA followed by Tukey’s post hoc test; means followed by the same letter do not differ significantly (p < 0.05). Alterations in the frequency of mitotic phases, particularly a reduction in anaphase/telophase and an accumulation of cells in prophase/metaphase, support the hypothesis that LiCl disrupts microtubule dynamics and delays mitotic progression.

Analysis of the cell cycle phases revealed (Fig. 4) no significant changes in the frequency of cells in prophase (χ^2^ Kruskal–Wallis_(5)_ = 7.18; *p* = 0.21) and telophase (χ^2^ Kruskal–Wallis_(5)_ = 13.02; *p* = 0.05), suggesting these stages were preserved. In contrast, significant alterations were observed in metaphase (χ^2^ Kruskal–Wallis_(5)_ = 27.21; *p* < 0.001) and anaphase (χ^2^ Kruskal–Wallis_(5)_ = 19.63; *p* < 0.01). A marked reduction in metaphase frequency was noted, with Treatment IV showing a complete absence of metaphase cells. Anaphase frequency increased in Treatments III (14.03%) and II (18.62%).

A plausible mechanism underlying this aneugenicity involves lithium chloride’s interaction with microtubule dynamics, particularly its influence on tubulin, a key component of the mitotic spindle apparatus. Previous studies indicate that lithium can alter tubulin polymerization and acetylation states, affecting spindle formation and stability during mitosis^40,41^. These microtubule-related disruptions are consistent with the aneugenic abnormalities observed, such as chromosomal lagging and spindle malfunctions. We conducted an in silico molecular modeling analysis to further investigate this hypothesis to explore the potential interaction between lithium ions and spindle microtubule fibers.

### Molecular modeling

**Fig. 5:**
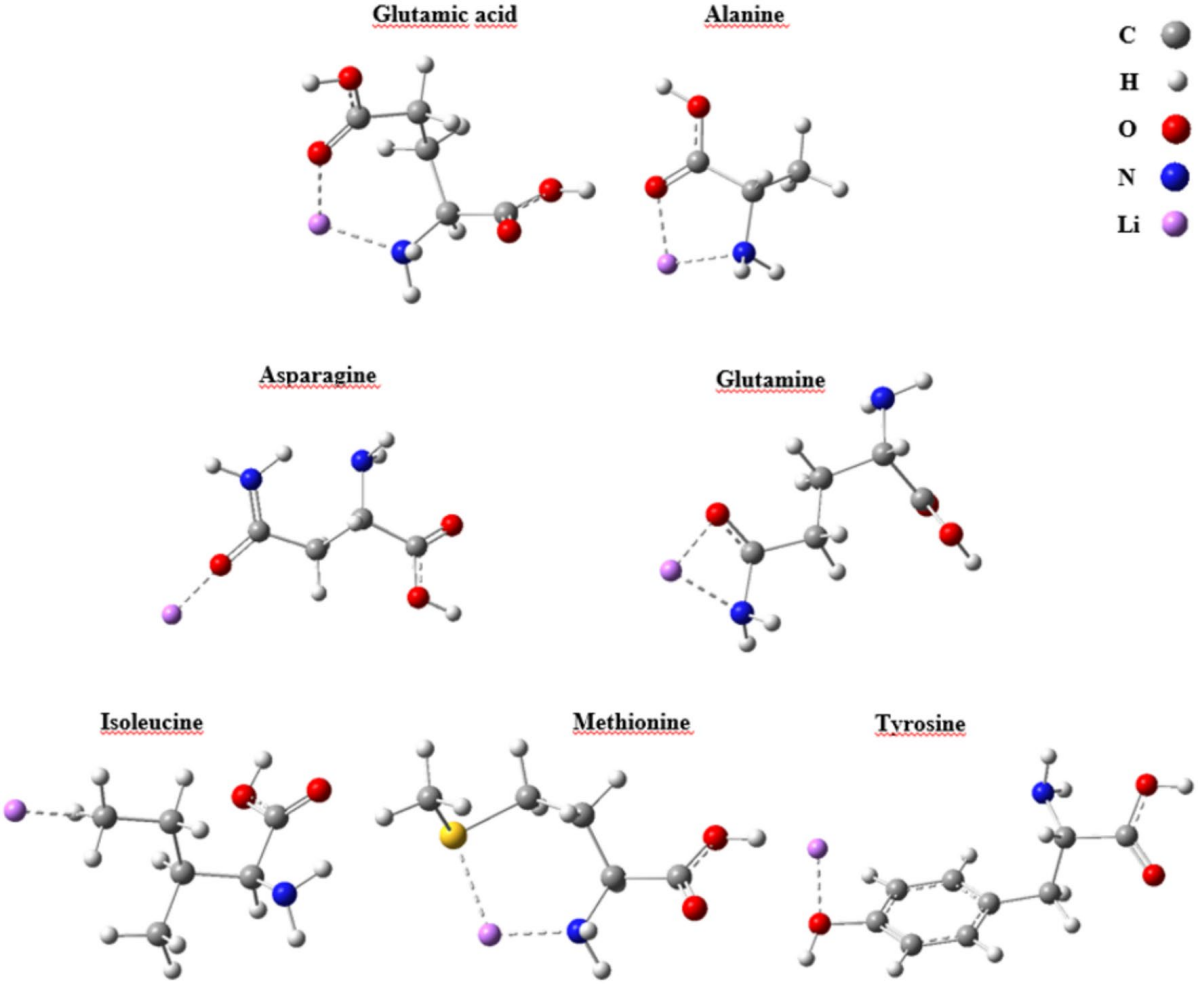
Predicted interaction sites between tubulin and Li^+^ ions. Molecular graphics illustrate the main amino acid residues located in the tubulin binding pocket that interact with Li^+^. The bond path and critical points of interaction are represented by dotted lines, highlighting coordination with negatively charged or polar side chains (e.g., Asp, Glu, and Ser residues). Atom colors: carbon (grey), oxygen (red), nitrogen (blue), lithium (purple). Structural analysis was performed using [insert method, e.g., molecular docking/QTAIM analysis], providing evidence that Li^+^ binds preferentially to electrostatically favorable sites.

Theoretical calculations suggested that the Li^+^ cation is capable of interacting with the side chains of several tubulin amino acid residues (Fig. 5). The QTAIM topological parameters—namely, electron density ($\rho (\mathbf{r})$), Laplacian of $\rho (\mathbf{r})$ ${\nabla }^{2}\rho$), kinetic energy density ($G(\mathbf{r})$), potential energy density ($v(\mathbf{r})$), and total electron energy density ($h(\mathbf{r})$)—are the main variables used to evaluate the characteristics of an interaction in the internuclear region. The values of $\rho (\mathbf{r})$ and ${\nabla }^{2}\rho$ provide information about the nature and strength of the interaction between two attractors at the bond critical point (BCP). Furthermore, the values of $h(\mathbf{r})$ help characterize the type of interaction. Values of $\rho (\mathbf{r})$ > 0.2 a.u. suggest *shared* interactions, such as covalent bonds. In this case, ${\nabla }^{2}\rho <0$ indicates that the $\rho (\mathbf{r})$ is concentrated at the BCP, and $h(\mathbf{r})<0$ denotes strong interactions. For values of $\rho (\mathbf{r})$ < 0.1 a.u., the interactions are classified as *closed-shell*, as observed in intermolecular interactions (e.g., hydrogen bond, van der Waals interactions). Here, ${\nabla }^{2}\rho >0$ indicates $\rho (\mathbf{r})$ depletion at the BCP, with the nuclear attractors (atomic nuclei) carrying most of the charge, and $h(\mathbf{r})>0$ suggesting weak interactions. Finally, 0.1 < $\rho (\mathbf{r})$  < 0.2 corresponds to interactions with a partially covalent character, where ${\nabla }^{2}\rho$ can be either positive or negative. In this case, $h(\mathbf{r})$ may also be positive or negative, but with low magnitude. The $|v(\mathbf{r})|/G(\mathbf{r})$ ratio further assists in characterizing the interaction: $|v|/G$ < 1.0 indicates a closed-shell interaction; $|v|/G$ > 2.0 corresponds to a shared interaction; and 1.0 < $|v|/G$  < 2.0 denotes a partially covalent interaction.

The topological parameters obtained indicated that Li^+^ ion interactions with amino acid side chains occur with relatively low $\rho (\mathbf{r})$ values (ρ < 0.1 a.u.) and are associated with ${\nabla }^{2}\rho > 0$. Moreover, the $|v|/G$ < 1.0 was for all cases, confirming that these interactions are *closed-shell* in nature—typical of electrostatic or ion–dipole interactions (Table 3).

**Table 3:** Topological parameters obtained by QTAIM and binding energies (BE), in kcal/mol, for the interactions between the Li^+^ cation and the side chains of selected tubulin amino acid residues. All values were computed at the M06-2X/6–311++ G(d,p) level of theory.

Interaction	${\rho }_{BCP}$^(*a*)^ (a.u.)	${\nabla }^{2}{\rho }_{BCP}$^(*b*)^ (a.u.)	$G(\text{r})$^(*c*)^ (a.u.)	$v(\text{r})$^(*d*)^ (a.u.)	$h(\text{r})$^(*e*)^ (a.u.)	$\frac{\left|v(\text{r})\right|}{G(\text{r})}$	BE (kcal/mol)
Li^+^ $\cdots$ glutamic acid							
Li^+^ $\cdots$ O–C (Glu)	0.03845	0.31354	0.06447	− 0.05055	0.01392	0.8	− 75.37
Li^+^ $\cdots$ N–C (Glu)	0.03314	0.20526	0.04425	− 0.03719	0.00706	0.8	
Li^+^ $\cdots$ alanine							
Li^+^$\cdots$ O–C (Ala)	0.03483	0.29477	0.05868	− 0.04367	0.01501	0.7	− 70.12
Li^+^ $\cdots$ N–C (Ala)	0.02853	0.18598	0.03792	− 0.02934	0.00858	0.8	
Li^+^ $\cdots$ asparagine							
Li^+^ $\cdots$ O–C (Asn)	0.04767	0.43243	0.08995	− 0.07178	0.01816	0.8	− 59.99
Li^+^ $\cdots$ glutamine							
Li^+^$\cdots$ O–C (Gln)	0.05030	0.39437	0.08488	− 0.07117	0.01371	0.8	− 54.11
Li^+^ $\cdots$ N–C (Gln)	0.03172	0.21386	0.04572	− 0.03798	0.00774	0.8	
Li^+^ $\cdots$ isoleucine							
Li^+^ $\cdots$ O–C (Ile)	0.02328	0.14383	0.03109	− 0.02623	0.00487	0.8	− 19.55
Li^+^ $\cdots$ methionine							
Li^+^ $\cdots$ S–C (Met)	0.02616	0.11535	0.02707	− 0.02530	0.00177	0.9	− 67.01
Li^+^$\cdots$ N–C (Met)	0.03524	0.21915	0.04746	− 0.04013	0.00733	0.8	
Li^+^ $\cdots$ tyrosine							
Li^+^ $\cdots$ C–C (Tyr)	0.03538	0.28751	0.05893	− 0.04598	0.01295	0.8	− 43.45

^a^Total electronic density on BCP.

^b^Laplacian of electron density on BCP.

^c^Lagrangian Kinect energy.

^d^Potential energy density.

^e^Total energy density.

Despite the absence of covalent character, the counterpoise-corrected binding energies are remarkably high. The interaction energies of Li^+^ with Glu200B (− 75.37 kcal/mol), Ala316B (− 70.12 kcal/mol), and Met (− 67.01 kcal/mol) are particularly significant and fall within the upper range reported for Li^+^ interactions with organic moieties such as carboxylates, amides, and hydroxyl-containing groups (typically − 20 to − 75 kcal/mol). These values are comparable to or even exceed those of model systems involving Li^+^–carboxylate complexes and Li^+^–amide interactions reported in the literature^42,43^.

These findings suggest that Li^+^ forms highly stabilizing interactions with specific residues in the tubulin binding site, most likely driven by strong electrostatic attraction, favorable spatial orientation, and possible partial charge delocalization. Notably, the strong interaction with Glu200B can be attributed to a negatively charged carboxylate group, which offers a high electron density region ideal for coordination with Li^+^. In the case of Ala316B, although its side chain is nonpolar, the interaction may involve nearby backbone carbonyl groups or be favored by geometric proximity within the binding pocket. Intermediate values observed for Asn167B (− 59.99 kcal/mol) and Gln (− 54.11 kcal/mol) likely result from interactions with polar amide groups, while the weak interaction with Ile378B (− 19.55 kcal/mol) is consistent with its aliphatic and hydrophobic character.

Previous studies have demonstrated that oxidative stress—a condition commonly associated with lithium exposure—can disrupt microtubule polymerization, impair spindle fiber assembly, and bypass mitotic checkpoints, ultimately leading to mitotic errors and chromosomal instability^44,45^. Consistent with this mechanism, we observed alterations in superoxide dismutase (SOD) activity in *Allium cepa* root meristems, with mean inhibition values of 15%, 12%, 21%, and 18% for Groups I, II, III, and IV, respectively. Although variance-based ANOVA did not reveal statistically significant differences among groups, the Jonckheere–Terpstra test detected a significant monotonic trend of increasing SOD inhibition with rising lithium concentrations (JT = 47, Z = 1.69, p = 0.046). Reduced SOD activity reflects a weakened primary antioxidant defense, leading to insufficient detoxification of superoxide radicals, accumulation of reactive oxygen species, and a heightened risk of oxidative damage to proteins, lipids, and DNA. Comparable findings have been reported in clinical studies, where lithium administration in healthy volunteers caused moderate reductions in SOD activity without major alterations in catalase activity or lipid peroxidation markers^46^. Taken together, these results indicate that lithium at environmentally relevant concentrations may elicit a mild but progressive oxidative challenge which, in combination with its direct interaction with tubulin, could impair spindle function through redox-sensitive mechanisms and reinforce an aneugenic mode of action.

## Conclusion

This study provides evidence that lithium chloride (LiCl), even at environmentally relevant concentrations, may induces mild oxidative stress, cytotoxicity, and genotoxicity in *Allium cepa* root cells. The lack of clastogenic effects, coupled with significant cytogenetic abnormalities and disturbances in the cell cycle, strongly supports an aneugenic mechanism of action. Our data suggest that lithium chloride interferes with mitotic progression primarily through its high-affinity interaction with tubulin, a key component of the spindle apparatus. This interaction likely impairs microtubule dynamics, disrupting chromosome segregation and spindle malfunction. Considering the increasing environmental release of lithium through industrial and pharmaceutical sources, these findings underscore the urgent need to assess the ecological implications of lithium-based compounds, particularly their potential to disrupt mitotic processes in non-target species.

## Data Availability

The datasets used and/or analyzed during the current study are available from the corresponding author (L.M.A., almeidalm@ueg.br) on reasonable request.

## References

[1] Bustamante, P., Bocher, P., Cherel, Y., Miramand, P. & Caurant, F. Distribution of trace elements in the tissues of benthic and pelagic fish from the Kerguelen Islands. *Sci. Total Environ.* **313**, 25–39 (2003). 12922058 10.1016/S0048-9697(03)00265-1

[2] Metian, M. et al. Trace element bioaccumulation in reef fish from New Caledonia: Influence of trophic groups and risk assessment for consumers. *Mar. Environ. Res.* **87–88**, 26–36 (2013). 23623270 10.1016/j.marenvres.2013.03.001

[3] Qin, G. et al. Soil heavy metal pollution and food safety in China: Effects, sources and removing technology. *Chemosphere* **267**, 129205. 10.1016/j.chemosphere.2020.129205 (2021). 33338709 10.1016/j.chemosphere.2020.129205

[4] de Souza, V. B., Hollas, C. E., Bortoli, M., Manosso, F. C. & de Souza, D. Z. Heavy metal contamination in soils of a decommissioned landfill southern Brazil: Ecological and health risk assessment. *Chemosphere* **339**, 139689. 10.1016/j.chemosphere.2023.139689 (2023). 37543230 10.1016/j.chemosphere.2023.139689

[5] Aral, H. & Vecchio-Sadus, A. Toxicity of lithium to humans and the environment—a literature review. *Ecotoxicol. Environ. Saf.* **70**, 349–356. 10.1016/j.ecoenv.2008.02.026 (2008). 18456327 10.1016/j.ecoenv.2008.02.026

[6] Krebs, R. E. Alkali earth metals: Periods 2 to 7, Group 2 (IIA). In *The History and Use of Our Earth’s Chemical Elements: A Reference Guide* 39–64 (Greenwood Press, 2006).

[7] Li, H., Eksteen, J. & Kuang, G. Recovery of lithium from mineral resources: State-of-the-art and perspectives: A review. *Hydrometallurgy* **189**, 105129. 10.1016/j.hydromet.2019.105129 (2019).

[8] Bibienne, T., Magnan, J. F., Rupp, A. & Laroche, N. From mine to mind and mobiles: Society’s increasing dependence on lithium. *Elements* **16**, 265–270. 10.2138/gselements.16.4.265 (2020).

[9] Flexer, V., Baspineiro, C. F. & Galli, C. I. Lithium recovery from brines: A vital raw material for green energies with a potential environmental impact in its mining and processing. *Sci. Total Environ.* **639**, 1188–1204. 10.1016/j.scitotenv.2018.05.223 (2018). 29929287 10.1016/j.scitotenv.2018.05.223

[10] Goonan, T. G. Lithium use in batteries. *U.S. Geological Survey Circular* **1371** (2012). https://pubs.usgs.gov/circ/1371/

[11] U.S. Geological Survey (USGS). *Mineral Commodity Summaries 2022*. (USGS, 2022). 10.3133/MCS2022

[12] International Energy Agency (IEA). *Energy Technology Perspectives 2017: Catalysing Energy Technology Transformations*. (OECD/IEA, 2017). https://iea.blob.core.windows.net/assets/a6587f9f-e56c-4b1d-96e4-5a4da78f12fa/Energy_Technology_Perspectives_2017-PDF.pdf

[13] Barbosa, H., Soares, A. M. V. M., Pereira, E. & Freitas, R. Lithium: A review on concentrations and impacts in marine and coastal systems. *Sci. Total Environ.* **857**, 159374. 10.1016/j.scitotenv.2022.159374 (2023). 36240931 10.1016/j.scitotenv.2022.159374

[14] Silva, G. A., Afonso, J. C. & Mahler, C. F. Lithium-ion batteries: Overview of resources and recycling processes. *J. Clean. Prod.* **208**, 1236–1254. 10.1016/j.jclepro.2018.10.168 (2019).

[15] Anderson, B. G. The apparent thresholds of toxicity to *Daphnia magna* for chlorides of various metals when added to Lake Erie water. *Trans. Am. Fish. Soc.* **78**, 96–113. 10.1577/1548-8659(1948)78[96:TATOTT]2.0.CO;2 (1950).

[16] Chandra, S. J., Kote, N. V., Sandya, S. & Sharath Chandra, S. P. Lithium nitrate induced biochemical modifications in *Catla catla* upon short term exposure. *Pharmacogn. J.* **12**, 1705–1709. 10.5530/pj.2020.12.230 (2020).

[17] Costa, R. S., Oliveira, P. & Guilhermino, L. Effects of nanoplastics, lithium, and their mixtures on *Corbicula fluminea*: Preliminary findings. *Adv. Sci. Technol. Innov.*10.1007/978-3-030-59320-9_57 (2021).

[18] Emery, R., Klopfer, D. C. & Skalski, J. R. *Incipient Toxicity of Lithium to Freshwater Organisms Representing a Salmonid Habitat* (Richland, 1981).

[19] Kszos, L. A., Beauchamp, J. J. & Stewart, A. J. Toxicity of lithium to three freshwater organisms and the antagonistic effect of sodium. *Ecotoxicology* **12**, 427–437. 10.1023/A:1026160323594 (2003). 14649425 10.1023/a:1026160323594

[20] Liu, D. et al. Lithium promotes the production of reactive oxygen species via GSK-3β/TSC2/TOR signaling in the gill of zebrafish (*Danio rerio*). *Chemosphere* **195**, 854–863. 10.1016/j.chemosphere.2017.12.130 (2018). 29291576 10.1016/j.chemosphere.2017.12.130

[21] Pinto Vidal, F. A. et al. Metamorphic acceleration following the exposure to lithium and selenium on American bullfrog tadpoles (*Lithobates catesbeianus*). *Ecotoxicol. Environ. Saf.* **207**, 111101. 10.1016/j.ecoenv.2020.111101 (2021). 32905937 10.1016/j.ecoenv.2020.111101

[22] Pinto-Vidal, F. A. et al. Effects of lithium and selenium in the tail muscle of American bullfrog tadpoles (*Lithobates catesbeianus*) during premetamorphosis. *Environ. Sci. Pollut. Res.* **29**, 1975–1984. 10.1007/s11356-021-15686-5 (2022). 10.1007/s11356-021-15686-5 34363154

[23] Kuloğlu, S. S. et al. Dose-dependent toxicity profile and genotoxicity mechanism of lithium carbonate. *Sci. Rep.* **12**, 13504. 10.1038/s41598-022-17367-1 (2022). 35931740 10.1038/s41598-022-17838-0 PMC9355992

[24] Aral, H. & Vecchio-Sadus, A. Lithium: Environmental pollution and health effects. In *Encyclopedia of Environmental Health* 116–125 (Elsevier, 2011). 10.1016/B978-0-444-52272-6.00531-6.

[25] Reimann, C. & de Caritat, P. *Chemical Elements in the Environment: Factsheets for the Geochemist and Environmental Scientist* (Springer, 1998).

[26] CONAMA. Resolução CONAMA n° 401 de 04/11/2008. Dispõe sobre os limites máximos de chumbo, cádmio e mercúrio para as pilhas e baterias comercializadas no território nacional e os critérios e padrões para seu gerenciamento ambientalmente adequado (Ministério do Meio Ambiente, 2008).

[27] Alam, Z. F., Riego, A. J. V., Samson, J. H. R. P. & Valdez, S. A. V. The assessment of the genotoxicity of e-waste leachates from e-waste dumpsites in Metro Manila, Philliphines. *Int. J. Environ. Sci. Technol.*10.1007/s13762-018-1719-6 (2018).

[28] Gichner, T., Patková, Z., Száková, J. & Demnerová, K. Cadmium induces DNA damage in tobacco roots, but no DNA damage, somatic mutations or homologous recombination in tobacco leaves. *Mutat. Res. Genet. Toxicol. Environ. Mutagen.* **559**, 49–57. 10.1016/j.mrgentox.2003.12.008 (2004). 10.1016/j.mrgentox.2003.12.008 15066573

[29] Türkoğlu, Ş. Determination of genotoxic effects of chlorfenvinphos and fenbuconazole in *Allium cepa* root cells by mitotic activity, chromosome aberration, DNA content, and comet assay. *Pest. Biochem. Physiol.* **103**, 224–230. 10.1016/j.pestbp.2012.06.001 (2012).

[30] Srivastava, A. K. & Singh, D. Assessment of malathion toxicity on cytophysiological activity, DNA damage and antioxidant enzymes in root of Allium cepa model. *Sci. Rep.* **10**, 886. 10.1038/s41598-020-57840 (2020). 31964992 10.1038/s41598-020-57840-y PMC6972773

[31] de la Roche, N. M. et al. Novel fragment-derived colchicine-site binders as microtubule-destabilizing agents. *Eur. J. Med. Chem.* **241**, 114614. 10.1016/j.ejmech.2022.114614 (2022). 35939994 10.1016/j.ejmech.2022.114614

[32] Hohenberg, P. & Kohn, W. Inhomogeneous electron gas. *Phys. Rev.* **136**, B864–B871. 10.1103/PhysRev.136.B864 (1964).

[33] Kohn, W. & Sham, L. J. Self-consistent equations including exchange and correlation effects. *Phys. Rev.* **140**, A1133–A1138. 10.1103/PhysRev.140.A1133 (1965).

[34] Zhao, Y. & Truhlar, D. G. The M06 suite of density functionals for main group thermochemistry, thermochemical kinetics, noncovalent interactions, excited states, and transition elements. *Theor. Chem. Acc.* **120**, 215–241. 10.1007/s00214-007-0310-x (2008).

[35] Bader, R. F. W., Nguyen-Dang, T. T. & Tal, Y. *A Topological Theory of Molecular Structure* (1981).

[36] Bader, R. F. W. & Essén, H. The characterization of atomic interactions. *J. Chem. Phys.* **80**, 1943–1960. 10.1063/1.446956 (1984).

[37] Van Duijneveldt, F. B., Rijdt, J. & van Lenthe, J. H. State of the art in counterpoise theory. *Chem. Rev.* **94**, 1873–1885 (1994).

[38] StatSoft Inc. *STATISTICA (Data Analysis Software System), Version 7.* (StatSoft Inc., 2004). https://www.statsoft.com

[39] Pastor, N., Kaplan, C., Domínguez, I., Mateos, S. & Cortés, F. Cytotoxicity and mitotic alterations induced by non-genotoxic lithium salts in CHO cells in vitro. *Toxicol. In Vitro* **23**, 432–438. 10.1016/j.tiv.2009.01.009 (2009). 19444924 10.1016/j.tiv.2009.01.009

[40] Gibbons, B. & Gibbons, I. Lithium reversibly inhibits microtubule-based motility in sperm flagella. *Nature* **309**, 560–562. 10.1038/309560a0 (1984). 6728011 10.1038/309560a0

[41] Bennett, G. S., Hollander, B. A., Laskowska, D. & DiLullo, C. Rapid degradation of newly synthesized tubulin in lithium-treated sensory neurons. *J. Neurochem.* **57**, 130–139. 10.1111/j.1471-4159.1991.tb02107.x (1991). 1675659 10.1111/j.1471-4159.1991.tb02107.x

[42] Luo, Y. R. *Comprehensive Handbook of Chemical Bond Energies* (CRC Press, 2007). 10.1201/9781420007282

[43] Skarmoutsos, I., Ponnuchamy, V., Vetere, V. & Mossa, S. Li+ solvation in pure, binary, and ternary mixtures of organic carbonate electrolytes. *J. Phys. Chem. C* **119**, 4502–4515. 10.1021/jp511132c (2015).

[44] Livanos, P., Galatis, B., Quader, H. & Apostolakos, P. Disturbance of reactive oxygen species homeostasis induces atypical tubulin polymer formation and affects mitosis in root-tip cells of *Triticum turgidum* and *Arabidopsis thaliana*. *Cytoskeleton* **69**, 1–21. 10.1002/cm.20538 (2012). 21976360 10.1002/cm.20538

[45] D’Angiolella, V., Santarpia, C. & Grieco, D. Oxidative stress overrides the spindle checkpoint. *Cell Cycle* **6**, 576–579. 10.4161/cc.6.5.3934 (2007). 17351333 10.4161/cc.6.5.3934

[46] Khairova, R. et al. Effects of lithium on oxidative stress parameters in healthy subjects. *Mol. Med. Rep.* **5**(3), 680–682. 10.3892/mmr.2011.732 (2012). 22200861 10.3892/mmr.2011.732 PMC3289682

